# A new method for obtaining the magnetic shape anisotropy directly from electron tomography images

**DOI:** 10.3762/bjnano.13.51

**Published:** 2022-07-05

**Authors:** Cristian Radu, Ioana D Vlaicu, Andrei C Kuncser

**Affiliations:** 1 National Institute of Materials Physics, Magurele, Romania; 2 Faculty of Physics, University of Bucharest, Bucharest, Romania

**Keywords:** electron tomography, magnetite, Python, shape anisotropy

## Abstract

A new methodology to obtain magnetic information on magnetic nanoparticle (MNP) systems via electron tomography techniques is reported in this work. The new methodology is implemented in an under-development software package called Magn3t, written in Python and C++. A novel image-filtering technique that reduces the highly undesired diffraction effects in the tomography tilt-series has been also developed in order to increase the reliability of the correlations between morphology and magnetism. Using the Magn3t software, the magnetic shape anisotropy magnitude and direction of magnetite nanoparticles has been extracted for the first time directly from transmission electron tomography.

## Introduction

For any nanoparticle (NP) system, among the most important pieces of physical information for scientists is information related to the morphology (size, shape, and organization) of its constituents. In nanoscale systems, this information becomes more important due to the potential presence of low-dimensionality effects. In the case of magnetic nanoscale systems, a complete knowledge of morphology is even more important because the configuration of magnetic moments in the system strongly depends on shape and size, through the shape anisotropy energy, and on the NP organization, through long-range dipole interactions. In other words, magnetic properties are strongly correlated with morphology. There are quite a few ways to provide primary morphological information on NP systems, each of them with specific strengths and weaknesses. A very brief review of such techniques is given in the following.

The particle size and shape of particulate systems ranging from the nanometer (characteristic to catalysis, sensoristics, spintronics, and some medical therapies [1–3]) over the micrometer (pharmaceutical industry and powder metallurgy) to the millimeter domain (mining and minerals) is one of the primary experimental information in materials science. The size of an entity is considered as the size of a well-defined, usually simple shape (rod, sphere, or ellipsoid). For the vast majority of systems, including the ones in our work, the size is reported under the assumption of a quasi-spherical shape of the constituent particles.

There is a multitude of techniques dedicated to nanoparticle analysis, which are able to provide information regarding the mean size and/or size distribution within a limited domain of sizes, for example, X-ray diffraction, magnetometry [4], transmission/scanning electron imaging [5–6], small-angle scattering techniques [7], dynamic light scattering, laser diffraction [8–9], resonant mass measurement, and spatial filter velocimetry. Each of these has certain features (i.e., ease of access, ease of use, suitability for particular systems, and global or local information), which limit their practical use to specific types of physical systems. Apart from direct imaging techniques (optical, scanning electron, and transmission electron microscopy), there are well-established indirect methods for the estimation of nanoparticle sizes. Usually, for such indirect methods, the envisaged NP system is assumed to have similar morphology for all constituents and a global estimation of NP size is “translated” from the interpretation of various manifestations of physical phenomena exploited by the investigation technique. The “translation” of the experimentally measured parameter to the parameter of interest (particle size in this case) is always accomplished by imperfect assumptions or extrapolations in the mathematical models. For example, in the case of X-ray diffraction, the mean crystal size is provided only for the volumes contributing to the diffraction pattern. The translation of the diffraction peak profile in terms of crystal size is provided by the Scherrer equation or by Rietveld analysis. Both of the models assume a homogeneous, well-defined shape of the nanoscale entities, which is almost always inaccurate. In the case of magnetometry, the size can be roughly evaluated from the magnetized volumes of the material [10] with a volume–size relationship depending on shape and morphology of the nanoparticles. Such indirect approaches more or less suit the investigated systems, and whether one technique is better than the other is difficult to reason in the absence of certain a priori knowledge of the system.

Direct imaging techniques provide the most accurate information regarding the morphology of a system, while direct and complete morphological information can be obtained only through 3D imaging [11]. Projection imaging is still the standard in morphological characterization but provides incomplete information. Although 3D imaging emphasizes the full morphology, most software provide generally volumes and surfaces of the investigated entities. The quantitative description of their morphology is considered mostly within the frame of morphometrics.

Regarding magnetic systems, a quantitative, simple evaluation of the shape is highly desired, which is able to offer to materials engineers a better understanding of the morphology–magnetism correlations within the system. For example, the size and shape analysis in 3D space could provide accurate data to be used as input in various physical models of magnetism (e.g., the micromagnetic Stoner–Wolfarth or Landau–Lifshitz–Gilbert models [12–13]). In the field of hyperthermia therapies [14–15], involving various types of ferrofluids, or in the development of rare earth-free permanent magnets [16–17], a more complex description of the morphology including particle shape and specific aspect ratio, as the main factors influencing the magnetic anisotropy [18–19] is absolutely necessary.

The new methodology that is proposed here, implemented in Magn3t, is addressing strongly desired morphology–magnetism correlations analyzing the shape anisotropy with respect to its magnitude, direction, and statistical distribution of MNPs within the system, starting from tomograms experimentally obtained on real MNP systems. The aspects addressed by the proposed software represent real challenges and have not been tackled before in such an interdisciplinary framework for MNP systems. On the one side, there are plenty of 3D analysis software programs available, which provide tools for investigating nanoscale morphological features in 3D data volumes (e.g., the very complex Avizo Amira or the more limited Tomviz and ImageJ, as well as software from TEM manufacturers, such as the visualizer Kay-JEOL). However, none of them is focused on answering the abovementioned issues, specific to magnetic nanoscale systems. On the other side, there are micromagnetic simulation software such as MERRILL [20], which can be used directly in correlation with an electron tomogram in order to give magnetic information [21]. However, they are not based on a general statistical distribution of morphological parameters relevant for magnetism. It is the task of this new proposed software to derive such a statistical distribution for morphological parameters relevant for magnetism and to use them in order to ultimately compute the magnetic energy of the MNP system with its most dominant (in zero or low applied field) shape anisotropy contribution.

Summarizing, our work aims to provide new tools for solving the following issues of the scientific community: (i) the commonly unknown real distribution of shape anisotropy energy in nanoparticulate magnetic systems and (ii) the catastrophic effect of diffraction contrast on tomography reconstruction. The work includes a description of the in-house designed software for image analysis (Magn3t) together with a brief description of a newly designed image filtering utility. Further, a validity check of the software on fully resolved (simulated) input data is shown. The efficiency of the software is finally shown on a real-life, magnetite nanoparticle system.

## Experimental

For the preparation of the magnetite MNPs, the following starting materials were used as purchased: FeSO_4_·7H_2_O (Merck, 99.5%), hexadecyltrimethylammonium bromide (CTAB, 99+%, Acros Organics), NaOH (puriss p.a., ACS reagent, reag. Ph. Eur., K < 0.02%, ≥98%, pellets), NH_4_OH (for analysis, 28–30 wt % solution of NH_3_ in water, Acros Organics). The preparation of the MNPs was performed by reverse precipitation, in which an aqueous solution (50 mL) of FeSO_4_ with the surfactant CTAB (2:1, mass ratio) was slowly dripped into a 50 mL basic solution of NaOH and NH_4_OH. The solution turned from transparent to a green blurred suspension and almost instantly to reddish color. The suspension was stirred at 70 °C for 5 h, which allowed for the formation of a uniform dark suspension in the whole reaction mass. The dark precipitate was separated by multiple subsequent cycles of centrifugation and washing with water, and it was air-dried at 70 °C overnight in an oven.

The experimental tomographic series was acquired with a JEOL 2100 transmission electron microscope (TEM). The instrument was operated at a voltage of 200 kV and the images have been obtained in scanning transmission electron microscopy (STEM) operation mode, using the high-angle annular dark-field (HAADF) detectors and an appropriate camera length. The TEM specimen was prepared by a standard powder method, using a 300 Mesh, lacey Carbon, Cu grid.

X-ray diffraction (XRD) has been performed on MNPs using a Bruker D8 Advance diffractometer. MAUD software has been used for Rietveld refinement.

## Software Description

Magn3t is using as main input data a previously 3D reconstructed volume of voxels. There is a wide range of solutions for 3D reconstruction of image series provided either for micro tomography or electron tomography (Avizo, ImageJ, IMOD, tomo3d, or Genfire). The proposed program was designed to work directly with electron tomograms reconstructed using Genfire [22]. Although Magn3t is technically capable to provide correct information regarding particle size distribution (with particle separation capabilities) for any 3D volume, the full power of the program is revealed for magnetic nanoparticulate systems.

The results provided by Magn3t starting from a reconstructed data volume are obtained performing the following tasks: (i) data volume thresholding, (ii) particle separation and identification, (iii) shape evaluation, and (iv) output of size, shape and shape anisotropy distributions. There is also (v) a filtering utility including a novel filter for image series, which might be very useful for reducing reconstruction artifacts. Each of the above mentioned aspects is briefly described in the following (more details can be found in Supporting Information File 1).

**Data volume thresholding** aims to find an optimal value according the which the data is separated in two classes (background and objects). More specifically, each voxel becomes 0 if its original value is lower that the threshold value, or 1 if the opposite is true. Two methods for thresholding have been implemented, an automated one using Otsu’s method [23] and a heuristic one, based on visual inspection of the result. While Otsu’s method is a well-established technique in image processing, the other method based on the visual inspection depends greatly on the user experience and can be involved with success in the approach of more challenging situations.

**Particle separation and identification:** A modified version of the priority-flood algorithm [24] has been used in order to distinguish particles that are in contact with each other. As a first step, the distance transform of the 3D image is computed, that is, each voxel takes the value of its Euclidian distance to the closest background voxel. An efficient method for obtaining an approximation for the Euclidian distance as described in [25] is implemented in Magn3t. It is based on two iterations in opposite directions through the data. Each voxel takes a new value according to the weighted values of the neighboring voxels that have been reached before. The values of the weights are chosen in such a way that a satisfactory approximation for the Euclidian distance is obtained. The as-obtained distance transform image has local maxima, which are situated in the centers of the nanoparticles. The positions of the local maxima are obtained using the morphological reconstruction method [26]. The morphological reconstruction can be viewed as repeated dilation operations on a marker image until its boundary becomes equal to that of a mask image. The mask is the distance transform of the 3D image and the marker is the same 3D image but with a constant value subtracted from each voxel. The difference between the original distance transform and the morphological reconstruction will be an image consisting only of the local maxima with a minimum height equal to the subtracted value. The local maxima are then labeled and used as seeds for the watershed algorithm [27]. Each particle is reconstructed filling the distance transform image from the voxel with the largest value down but maintaining the labeling at the contact between adjacent particles.

**Shape evaluation:** The procedure consists of fitting each separated particle with an ellipsoid and recovering information about size, axis ratios, and orientation of the long axis from the fit. Both the direction and the magnitude of the semi-axes describing the fitting ellipsoids are extracted from the eigenvalues and, respectively, eigenvectors of the inertia tensor (see Supporting Information File 1). This method works under the assumption that the MNPs do not deviate too much from the most general ellipsoid shape with three different rotation axes. This assumption is usually valid for the vast majority of MNPs used in practice.

**Output size, shape, and anisotropy distributions:** A file containing MNP volume, position of its center of mass, magnitude and orientation of the three semi-axes under the ellipsoidal shape approximation for all MNPs is generated.

**Figure 1:**
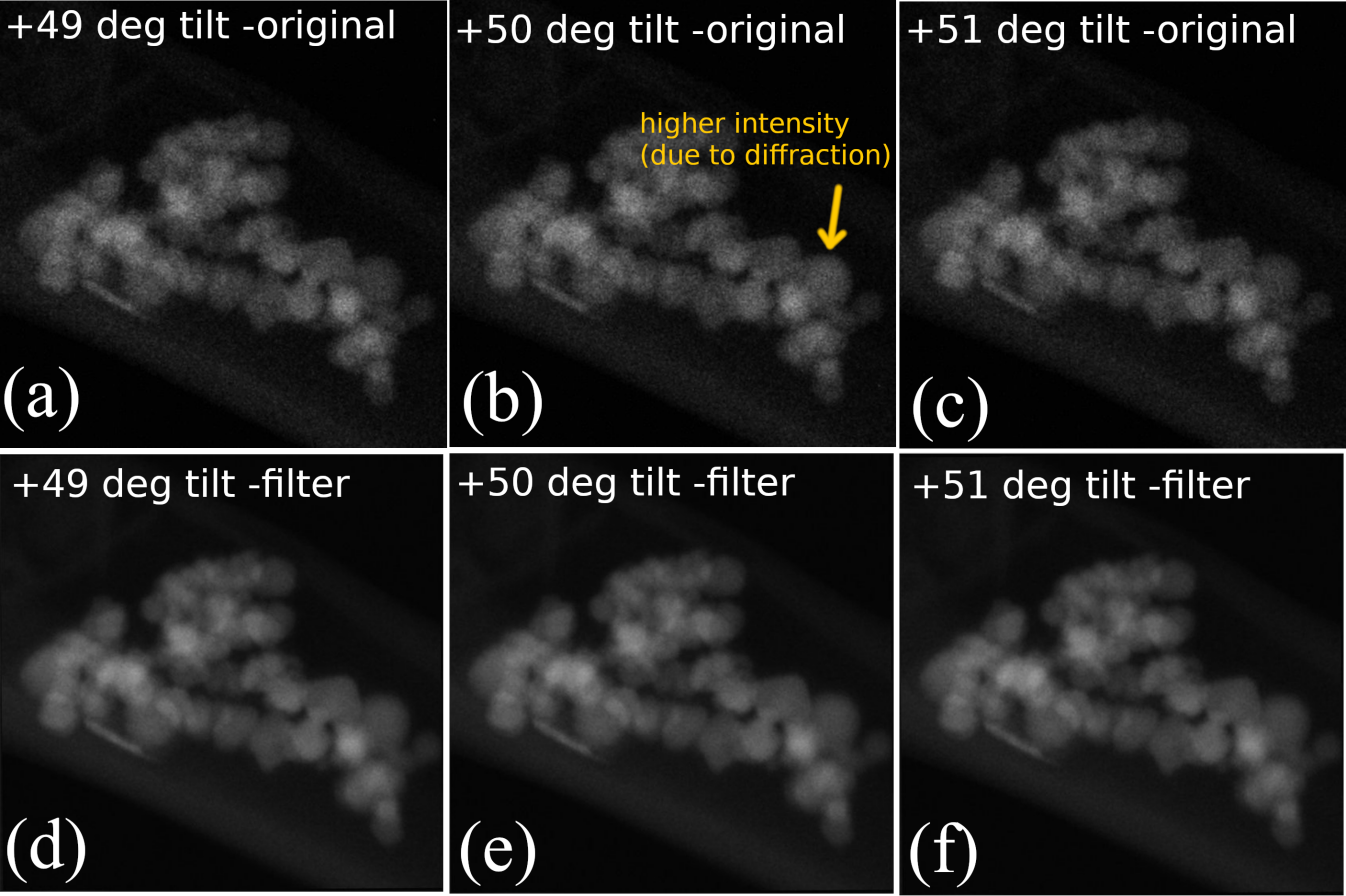
Typical example of image series filtering using the proposed method. Three successive images are presented before (a–c) and after filtering (d–f).

**Filtering utility:** Handling the diffraction contrast in electron tomography is still an ongoing issue [28–29]. In this regard, the Magn3t software is accompanied by an in-house developed novel image filtering tool, which can be applied on well-aligned experimental tomography tilt series, prior to the tomographic reconstruction. The filtering aims to reduce the diffraction contrast, a highly undesired effect in the tomography field, thus improving the quality of the tomographic reconstruction. The filtering strategy is based on the following two observations: (i) If the step dθ of the tilt series is small enough, provided a sufficiently simple system, there is no major difference between images obtained at angle θ and angle θ + dθ, and (ii) diffraction contrast is highly directional so it “triggers” in a very narrow interval around angle θ. Regarding the applicability of the filtering method, it is worth mentioning that even at a standard step size of 1°, the differences between images at successive angles of a very complex system might become unpractically large. In contrast, in order to reduce the differences between successive images, the step size may become unpractically low. The algorithm is based on an iterative averaging of images within the tilt series in order to reduce both noise and highly directional effects at the expense of a small decrease in the resolution (2–3% for 1° steps of the tilt series during the first iteration). The images (slices) of the specimen obtained at angles θ, θ − dθ and θ + dθ are averaged pixel-wise. The process can be repeated more than once if necessary, keeping in mind the decrease in the resolution. In Figure 1, an example of three successive images from a tomographic image series (of magnetite nanoparticles) before and after the applied filter is shown.

Several effects of the filter can be noticed in Figure 1. The most obvious effect is a noise removal of the images. The second one, which is little bit more difficult to observe, is the removal of the undesired “shining” of one of the NPs (Figure 1b) due to diffraction effects. The removal effect is very easy to observe in a dynamical manner, that is, while continuously looping the images. The last effect is related to a very slight decrease in the sharpness of NP edges. A difference-based representation of Figure 1 is shown in Supporting Information File 1. As with any image filter, the efficiency will strongly depend on the system complexity and user appeal. However, in this particular case, the filter had an obvious beneficial effect on the image series. Improvement in the quality of image series by de-noising and removal of diffraction effects translates into a better 3D reconstruction. The filtering utility can be used independently from the Magn3t software on any aligned experimental tilt series in the common mrc format.

## Results and Discussion

In order to demonstrate both capabilities and limits of the software, two examples are considered, that is, (i) an ideal case involving a simulated, fully described 3D volume containing partly overlapping ellipsoids and (ii) a 3D volume as obtained from reconstruction of an experimental tilt series. The ideal case with the simulated 3D volumes was considered important for a quality check of the program by observing the difference between the output parameters and the fully characterized input data. Experimental data obtained on magnetite NPs was used to check the software in a real-life situation.

### Theoretically generated system

A test volume filled with ellipsoids has been theoretically generated and analyzed. The ellipsoids have been generated with randomly distributed volumes (according to a normal distribution), shapes, and orientations. The ellipsoids are characterized by the semi-axes *a*, *b*, and *c*, of which *c* has always the highest value and is considered to coincide with the easy axis of magnetization [30]. The shape has been manipulated via the ratios *c*/*a* and *c*/*b*.

The test volume was initially a 3D matrix filled with values of 0. Each time an ellipsoid was generated, its occupied volume was filled with a value of 1, and the parameters of the generated ellipsoid were added to a list (reference data). The 3D matrix was saved in a mrc file in order to mimic a real tomogram. The mrc test volume was imported into Magn3t, and the obtained list of parameters was compared with the reference data.

**Figure 2:**
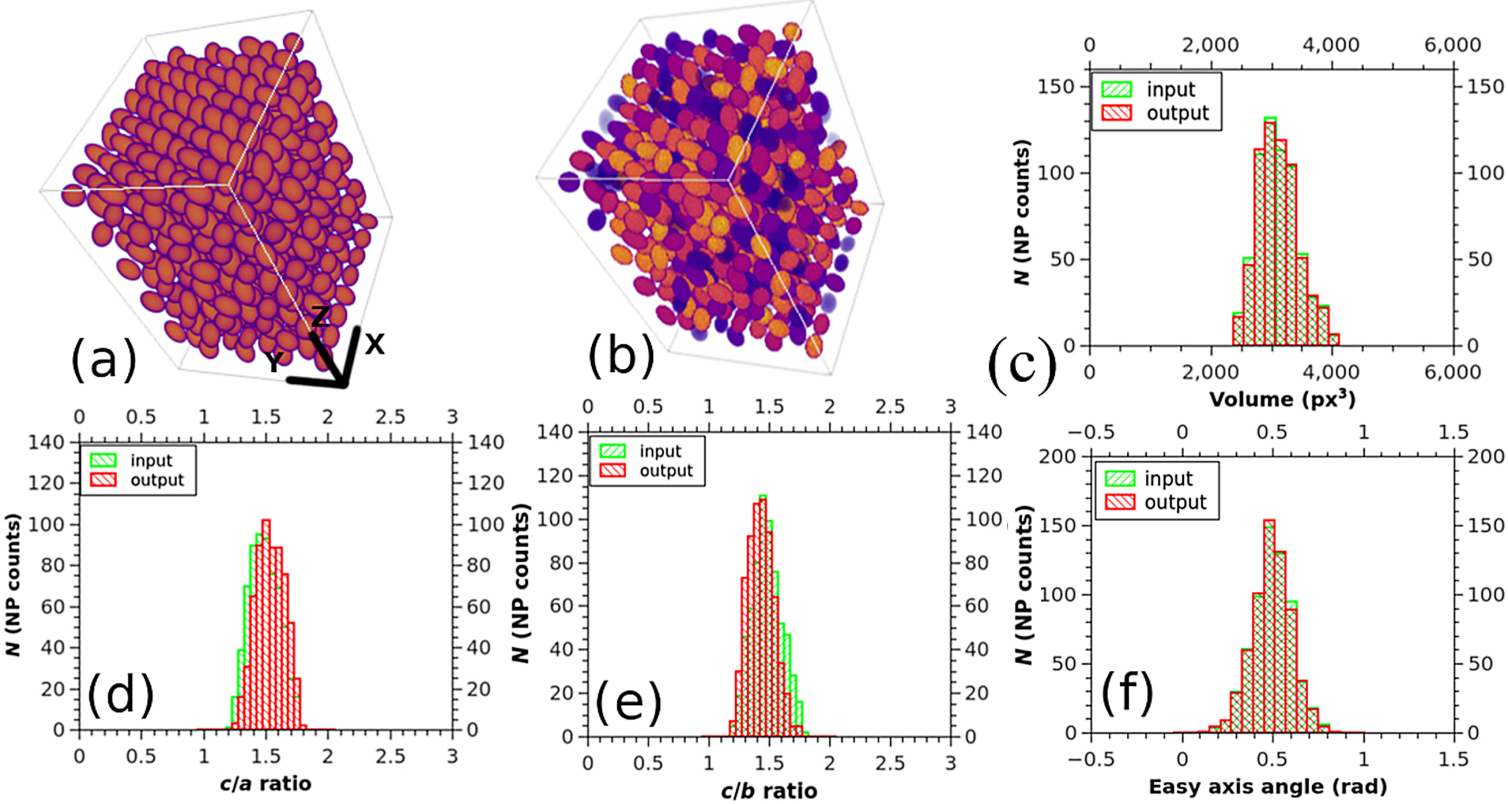
Simulated data volume (a), segmented data volume (b), and different outputs of Magn3t (c–f).

The simplest case with non-overlapping entities has been used as an initial test of the software. The results of the particle identification by the Magn3t software is shown in Figure 2b. The computed volume distribution as well as information about ellipsoid semi-axes (Figure 2c–f, red histograms) are presented in comparison with the reference data (Figure 2c–f, green histograms). The NP counts in each graph stand for the number of identified nanoparticles. While there is a very good agreement between the simulated and computed distributions of the volumes and easy-axes orientations, there is a ca. 6% shift in the semi-axis ratio distributions (Figure 2d for *c*/*a* and Figure 2e for *c*/*b*). This is a combined effect of the morphological reconstruction and of the poor resolution of the test volume, which affects mainly the semi-axes magnitude of the fitting ellipsoid. The higher the resolution, the lower the shift, but the higher the hardware requirements.

**Figure 3:**
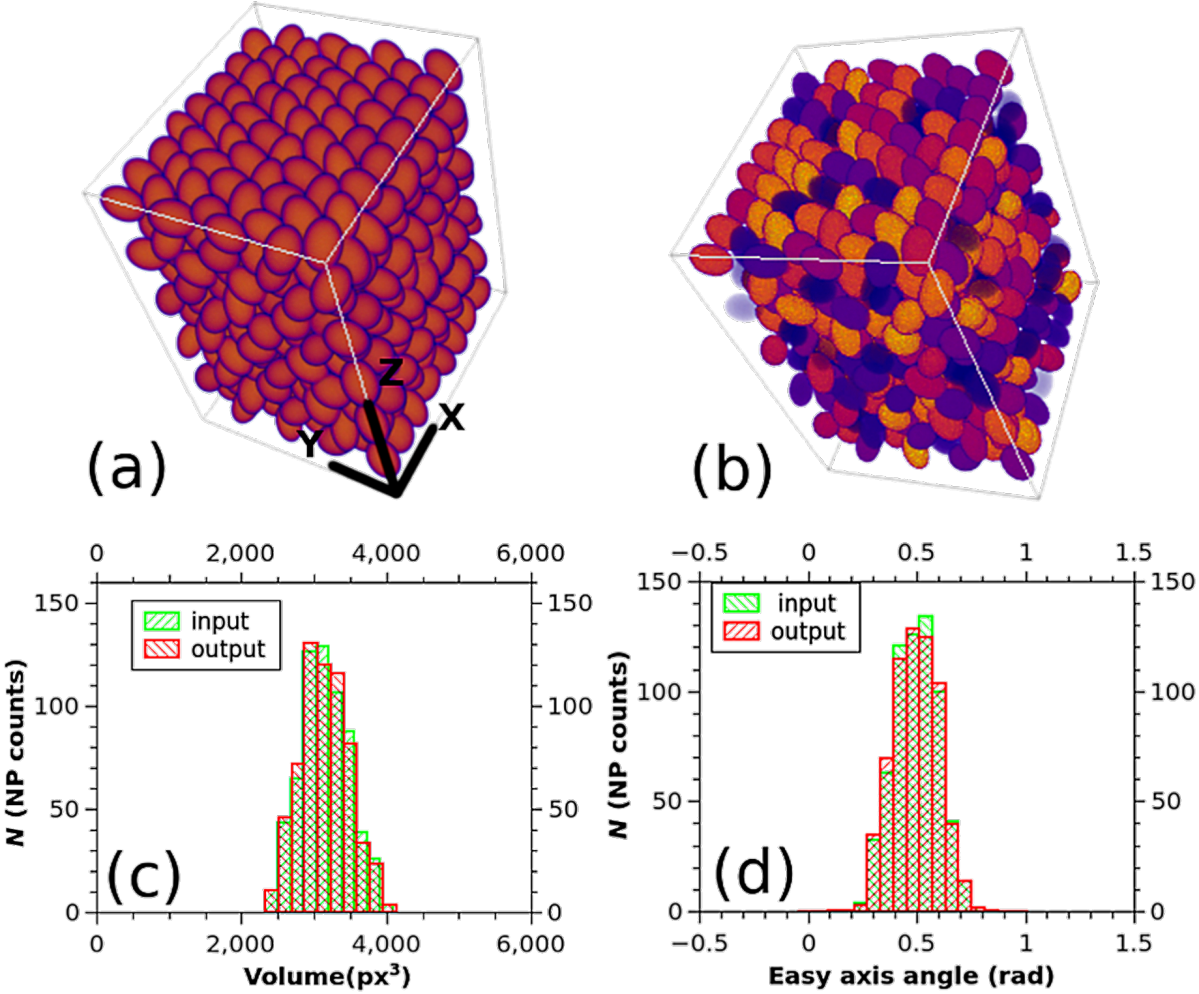
Simulated data volume (a), segmented data volume (b), different outputs of Magn3t (c, d).

Overlapping entities can be also managed by the software. Regarding this, a test data volume filled with ellipsoids has been generated (Figure 3). Again, size, shape, position, and orientation of the ellipsoids have a Gaussian distribution in space, this time without the constraint of no overlap.

### Experimentally obtained system

**Figure 4:**
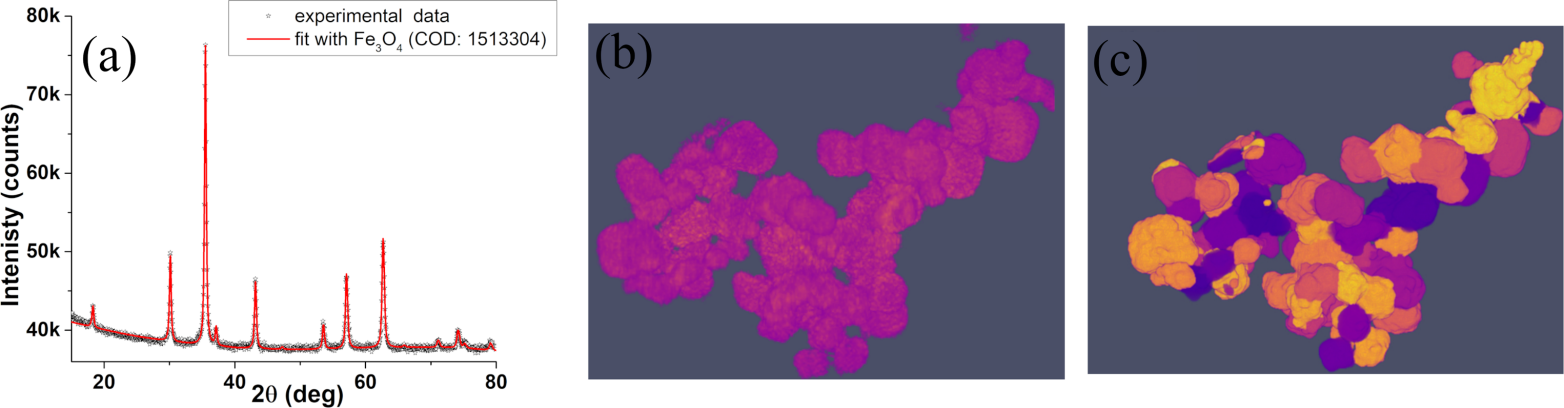
XRD pattern of Fe_3_O_4_ MNPs and associated Rietveld refinement (in red) (a). Tomviz perspectives on experimental tomogram data as obtained by Genfire (b) and tomogram data after segmentation and particle separation using Magn3t (c). Each of the identified particles is filled with a computer-generated color, just to visually differentiate between the particles.

A system of iron oxide nanoparticles has been used for further tests. A −67 to +67 tomographic tilt series has been obtained with 1° steps. The series has been aligned using Tomviz [31] and ImageJ [32] software. The aligned series has been reconstructed using Genfire. The Rietveld refinement of the XRD spectra (Figure 4a) revealed MNPs with a diameter of roughly 47 nm and with the Fe_3_O_4_ crystal structure. In addition to the XRD, electron tomography showed that the size of MNPs is indeed in the 40 nm range, but they have a slightly elongated morphology (Figure 4). For a quantitative description of the shape and extraction of the magnetic shape anisotropy from electron tomography results, the Magn3t program was used.

**Figure 5:**
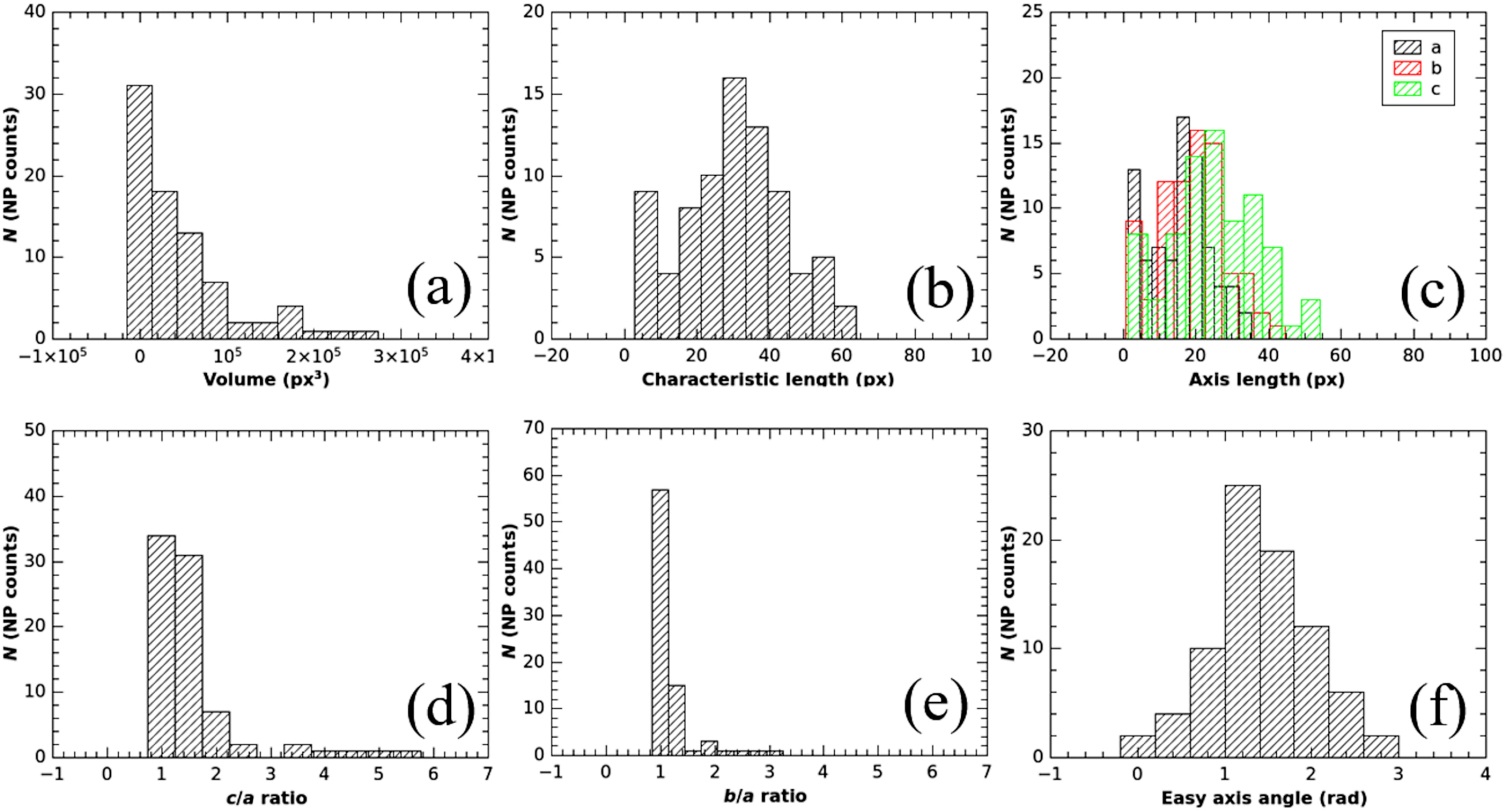
Different outputs of Magn3t algorithms, applied on experimentally obtained electron tomograms: volume distribution (a), characteristic length (b), long axis length (c), *c*/*a* and *b*/*a* ratios (d, e), easy axis angle (f), and estimation of demagnetization factors (g). Each pixel is 1.47 nm.

Quantitative information regarding size, shape, and orientation of the MNPs has been obtained using Magn3t. Figure 5c shows a higher average of dimensions along one reference axis as compared to the average dimensions along the other two orthogonal directions. The *a* and *b* axes distributions overlap, in accordance with *b*/*a* ratio having an average close to 1 (Figure 5f). These facts combined with the distribution of the *c*/*a* ratio (Figure 5d) state that the system consists of slightly elongated nanoparticles with almost circular transversal cross section. Using a calibration of 1.47 nm/px, it can be estimated that the MNPs have a cross section with an average diameter of roughly 30 nm whereas their length is, on average, slightly above 40 nm, which is compatible with the XRD measurements.

Orientation data is given in Figure 5g and points out that, on average, the nanoparticles are in the natural orientation, that is, the *c*-axis is close to the *c*-membrane plane. Starting from the *c*/*a* and *b*/*a* axis distributions provided by Magn3t, the demagnetization factors and the shape anisotropy energy [33–34] have been evaluated using the equations described in [35] and considering a saturation magnetization of 4·10^5^ A·m^−1^. An estimation of the magnetic shape anisotropy energy has been computed for each of the MNPs in the usual way, by taking the difference between the demagnetization energy of a MNP with spins oriented along the easy axis (highest demagnetization factor) and the demagnetization energy of the same MNP with spins oriented along the hard axis (lowest demagnetization factor). The automatic evaluation of the absolute value of shape anisotropy energy density (J·m^−1^) will be soon implemented in the software.

**Figure 6:**
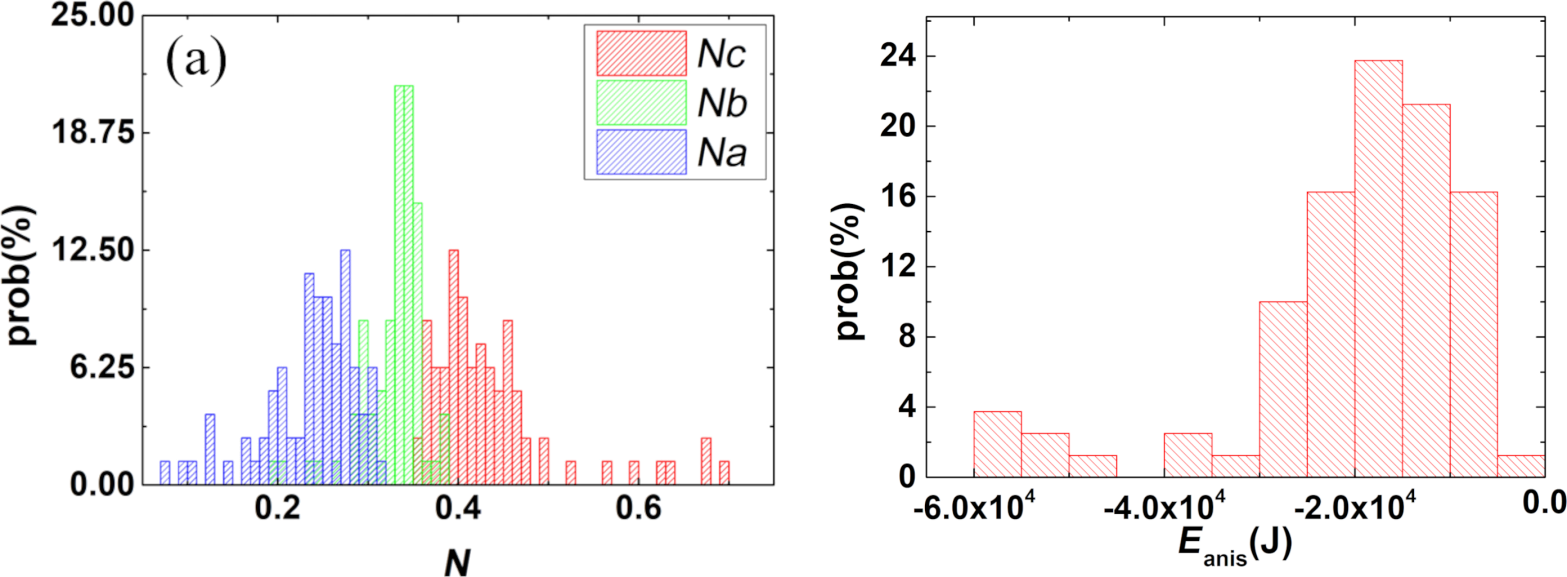
Histograms showing demagnetization factors (a) and shape anisotropy energy density (b) as obtained using the output of Magn3t.

Based on the complete morphological characterization of the system via transmission electron tomography and an a priori knowledge of the magnetization, statistical information regarding the orientation of MNPs (Figure 6a) as well as their shape anisotropy (Figure 6b) has been obtained. This kind of statistical information offers a valuable insight on any MNP system and, to the knowledge of the authors, there is no other technique capable of providing it.

While the proposed methodology and associated software is able to provide statistical anisotropy information, its applicability goes beyond magnetic nanoparticle systems. In fact, regardless of the magnetic shape anisotropy, the output information regarding nanoparticle position, volume, and/or shape (via ellipsoid fitting) might be useful in other situations involving physical systems composed of more or less dispersed entities, including porous systems. For example, by exploiting the relative distances between entities, some information about geometrical organization of a system can be derived. Estimating the differences between the fitted ellipsoid nanoparticles and the actual nanoparticles can provide additional information about the shape of the system entities. Using the abovementioned information as data input in artificial intelligence systems, such as neural networks, in order to identify and/or predict materials with special properties, should be explored.

## Conclusion

Magn3t software aims to provide a free, open source solution for the most frequent issues in magnetic nanoparticulate systems, that is, the evaluation of particle volume as well as the anisotropy magnitude and orientation for each entity of the system. It is dedicated to the improvement of both engineering and modeling of nanoparticulate systems, which are essential for advancements in medical fields (hyperthermia and drug delivery), permanent magnet industry, sensoristics, and spintronics.

The program has been tested not only on simulated tomograms but also on tomograms obtained on real-life systems of magnetite MNPs, obtained in-house by co-precipitation methods. Starting from the experimental data obtained using transmission electron microscopy, Magn3t has been used to identify and separate nanoparticles and fit them using ellipsoids. Within this framework, that is, the approximation on MNPs with ellipsoids, the morphology of MNPs has been characterized and statistical distributions on the orientation and size of the elliptical axes have been provided. Starting from the statistical data on the elliptical axes, the distribution of shape anisotropy energy density within the system has been computed.

The information provided by the software is suitable for the use in various magnetic/micro-magnetic models. In particular, the absolute value of shape anisotropy energy, evaluated based on elliptic axis ratios, is a valuable magnetic information that can be conveniently used in correlation with magnetometry measurements for a better understanding of the investigated system.

The utility for filtering images from a tilt series prior to 3D reconstruction has been shown to improve the quality of 3D reconstruction of a magnetite MNPs system. Thus, it is of potentially high interest in the field of electron tomography, where the diffraction contrast is highly undesired and, in many cases, not completely avoidable.

## Supporting Information

Additional technical information regarding the algorithms included in the Magn3t software. All source code is available on github (https://github.com/rdcrs/magn3t).

File 1 Magn3t algorithm details.

## Data Availability

All data will be made available upon reasonable request. Also, the source code of Magn3t can be found on github (https://github.com/rdcrs/magn3t).
