# Supplementary material for: A new method for obtaining the magnetic shape anisotropy directly from electron tomography images
Source: Beilstein J Nanotechnol. 2022 Jul 5;13:590–8. doi: 10.3762/bjnano.13.51 (PMC9273981; doi:10.3762/bjnano.13.51)
Supplement: File 1 — Magn3t algorithm details. [file Beilstein_J_Nanotechnol-13-590-s001.pdf]

BEILSTEIN JOURNAL OF NANOTECHNOLOGY

Supporting Information

for

A new method for obtaining the magnetic shape anisotropy directly from electron tomography images

Cristian Radu, Ioana D. Vlaicu and Andrei C. Kuncser

Beilstein J. Nanotechnol. **2022,** *13,* 590–598. doi:10.3762/bjnano.13.51

Magn3t algorithm details

License and Terms: This is a supporting information file under the terms of the Creative Commons Attribution License (https://creativecommons.org/licenses/by/4.0). Please note that the reuse, redistribution and reproduction in particular requires that the author(s) and source are credited and that individual graphics may be subject to special legal provisions.
The license is subject to the *Beilstein Journal of Nanotechnology* terms and conditions: (https://www.beilstein-journals.org/bjnano/terms)

Data volume thresholding aims to find an optimal value according the which the data is separated in two classes (background and objects). This is performed using Otsu's method, which reduces the problem to that of maximizing the inter-class variance:

$$\sigma^2(t) = \omega_0(t)\omega_1(t)[\mu_0(t) - \mu_1(t)]^2$$

where:

$$\omega_0(t) = \sum_{i=0}^{t-1} p(i)$$

$$\omega_1(t) = \sum_{L-1}^{i=t} p(i)$$

are the probabilities of class occurrence and

$$\mu_0(t) = \frac{\sum_{i=0}^{t-1} ip(i)}{\omega_0(t)}$$

$$\mu_1(t) = \frac{\sum_{i=t}^{L-1} ip(i)}{\omega_1(t)}$$

are the class averages.
It assumed that the data is represented as gray levels, with *p(i)* the normalized histogram.

A distance map is an image where each object voxel is labeled with the distance to its closest background voxel. It is obtained using a technique called weighted-distance transform, which is computed in two passes. The image is inspected from left to right, top to bottom, and front to back during the forward inspection and from right to left, bottom to top, and back to front during the backward inspection. The forward inspection mask is made of the voxels in the neighborhood of the current voxel that have not yet been reached, and the backward inspection mask is made of the voxels which have been already reached. In Figure S1, the voxels involved in the two inspections are shown. Underlined voxels are used in the backward inspection mask and the remaining voxels in the forward inspection mask. The weights in the mask are added to the temporary distance labels of the voxels. The weights are chosen such that a good approximation to the Euclidian distance is obtained. The computation of the distance map of an image I can be summarized by the following pseudo-code:

```
for z = 1 to n
  for y = 1 to n
    for x = 1 to n
      I (x, y, z) = min (i, j,k)∈fwd (I (x + i, y + j, z + k) + w(i, j, k))

for z = n downto 1
  for y = n downto 1
    for x = n downto 1
      I (x, y, z) = min (i, j,k)∈bwd (I (x + i, y + j, z + k) + w(i, j, k))
```

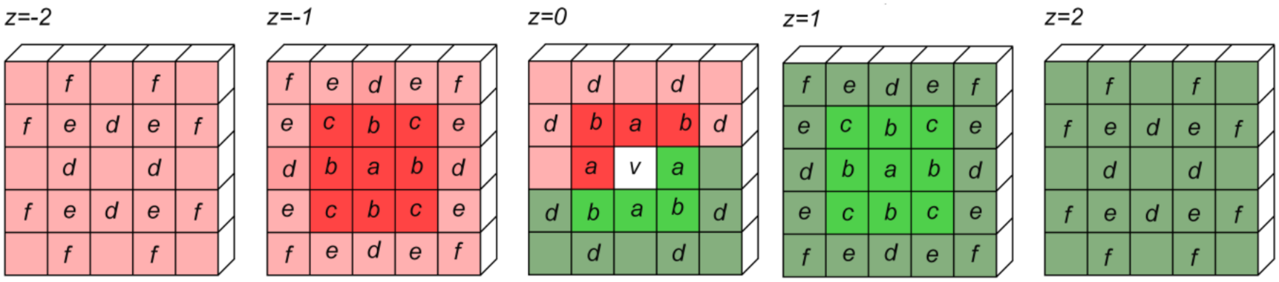

Figure S1: The 5 × 5 × 5 neighborhood showing the backward mask (dark red and light red) and the forward mask (dark green and light green). The letters “a” to “f” represent weights.

Particle separation is done using watershed-segmentation. It uses the distance transform of the object and a seed image, which consist of the local maxima of the distance map that should represent the center region of the particles. The segmentation is achieved using a priority-flood algorithm. Priority-flood is a depression-filling algorithm, which means that it fills an elevation image starting from seeds located at the lowest points. Our code uses a modified version that does the opposite, that is, it fills the image (which is the distance map of the object) starting at seeds located at the maxima of the distance map, that is, near the centers of the particles which make up the object, and going downwards. Initially, each seed has been labeled. The algorithm relies on the use of a priority queue, which is an ordered queue such that the voxels with the highest value are processed first and then removed from the queue. The pseudo-code of the procedure is presented below:

Let Q be a priority queue
Let Closed have the same dimensions as I_{DM} (distance map image)
Let Closed be initialized to false
Let S be the seed image

```
for all c in S do
    push c onto Q with priority I_DM (c)
    Closed(c) ← true

while Open is not empty do
    c ← pop(Open)
        for all neighbors n of c do
            if Closed(n) == false
                I_DM(n) ← min(I_DM(n), I_DM(c))
                Closed(n) ← true
                Push n onto Open with priority I_DM (n)
return I_DM
```

The returned data now has each particle of the object labeled differently.

The shape and orientation evaluation procedure consist of fitting each separated particle with an ellipsoid and recovering information about size, axis ratios, and orientation of the long axis from the fit. The fit procedure is performed as follows: First the center of mass of each particle is found. Then the inertia tensor is calculated using the following the expression:

$$I_{ij} = \frac{1}{V}\int r_i r_j dV$$

where r_i are x, y, z coordinates of each voxel with respect to the center of mass.

The eigenvalues and eigenvectors of the inertia tensor are the found:

$$\boldsymbol{Iv} = \lambda \boldsymbol{v}$$

While the directions of the ellipsoid semi-axes are along the eigenvectors, their magnitude is related to the eigenvalues by the following relation:

$$A_i = \sqrt{5\lambda_i}$$

Filtering utility

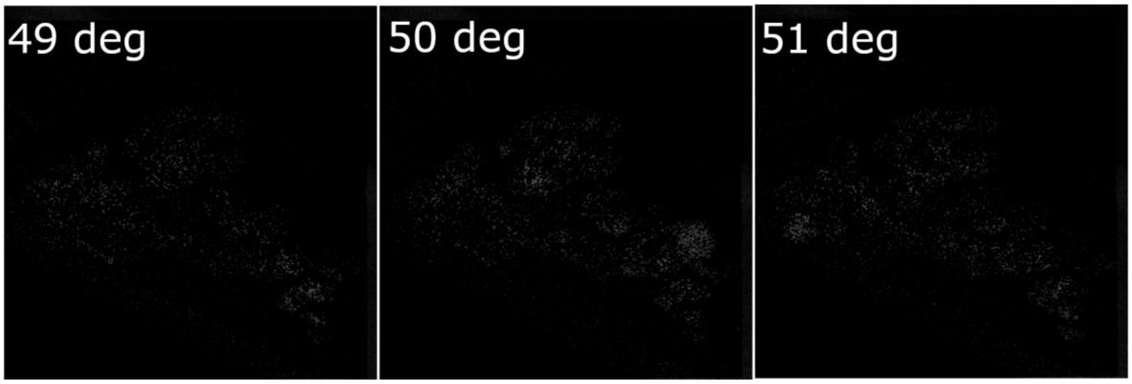

Figure S2: Images obtained by subtracting a filtered image from an unfiltered image @ 49°, 50° and 51° tilt angles, respectively.
